# Therapeutics

**Published:** 1879-04

**Authors:** 


### THERAPEUTICS.

JABORANDI IN PLEURITIC EFFUSIONS. — A. Laramée. (*L'Union Médicale du Canada*, January, 1879.)

Before coming to the main point, the author gives a brief history of this medicine. Jaborandi (*pilocarpus pinnatus*) is a shrub, 8 or 10 feet in height, with leaves alternate, pennate, compound, growing in the north of Brazil, near Pernambuco. It bears flowers and fruit, but up to the present, only the leaves, it seems, have been experimented with in therapeutics.

The active principle of jaborandi, pilocarpine, was discovered by Hardy in 1875. This alkaloid, known under the name of nitrate or muriate of pilocarpine, is high priced, but on the other hand an exceedingly small quantity, three centigrams, equals four grams of the leaves in infusion. The most common and economical plan of giving jaborandi is to give the infusion of the leaves, which is prepared by infusing four grams of the bruised leaves in a cup of boiling water for quarter of an hour, and give to the patient immediately afterward.

It is also given in the form of fluid extract in the dose of 30 drops to 4 grams, and in pills of 20 centigrams, each pill equaling 4 grams of the leaves. The muriate and nitrate of pilocarpine are often used for hypodermic injections in the proportions of 16 milligrams.

All the doses mentioned may be increased. The leaves furnish a large quantity of essential oil, but it has not yet been experimented with.

General opinion attributes the discovery of jaborandi to Dr. Continho, of Pernambuco. This gentleman having experimented with it, and having recognized sialagogues and sudorific properties in a high degree, sent some samples to France in 1873.

The contradictory reports, which have circulated for some time, regarding the effects of jaborandi arise from the fact that a plant coming from South America and bearing the same name was confounded with the true plant, but it is now certain that the true species is the only one exported to Europe and the United States.

Prof. Gubler, at the Beaufon Hospital, Paris, experimented with it on a large scale, and some months later, MM. A. Robin, Vulpian, Hardy and several others carefully studied jaborandi, especially its physiological properties. All recognized in it a powerful action on the salivary and sudoriferous glands. Since then jaborandi is regarded as the most energetic sudorific and sialagogue with which we are acquainted.

Under the action of this medicine, five to fifteen minutes usually, but never later than twenty-five minutes after its administration, salivation and sweating take place and go on increasing. Salivation, which begins first, ceases generally at the end of two hours, and the sweating a little later. The patients are tormented by a continual spitting, and frequently by nausea, and even vomiting, especially if they swallow the saliva. The salivary glands are then more or less swollen and at times painful. Swéating is less marked in persons subject to gastric disorders and constipation, but more free in those subject to sweating.

The temperature of the body is not sensibly modified. Sometimes vertigo comes on, but it is only temporary.

M. Gubler has noticed secondary actions, such as hypersecretion, from the lachrymal glands, from the nasal membrane and from the pharyngeal, tracheal and bronchial glands.

M. A. Robin observed that the day of the experiment the urine diminished notably, but the day after there was a considerable increase. However, other things being equal, it may be said that the reduction of the amount of urine is not proportioned to the loss of fluids from the skin and salivary glands.

There is a marked antagonism between jaborandi and atropine. At the University College, London, they succeeded with 6 milligrams of nitrate of pilocarpine in producing an abundant salivation, and with 3-10 of a milligram of atropine it was arrested. At Paris, M. Vulpian succeeded in preventing salivation and sweating in one of his patients, to whom he had given a few minutes previously a milligram of sulphate of atropine.

One drop of solution of nitrate of pilocarpine (in the proportion of 65 milligrams to 30 grams of water) is sufficient to produce contraction of the pupil when dropped in the eye.

The above are the most important physiological properties of jaborandi. The author gives the two following cases to illustrate its action in pleuritic effusions.

The first case, in December, 1877, was that of a man aged 40 years, the subject of pleurisy for about 12 days. All the signs indicating inflammation of the left pleura were present; pain in the side, dry cough, early effusion, producing dullness, which changed its position with that of patient, absence of respiratory murmur, bronchial respiration, etc. He had recourse first to the classic treatment, emetic, vesicants, calmatives, etc. He finally tried jaborandi, 4 grams of the leaves infused for 15 minutes and given in one dose. Five minutes after, salivation began and lasted for nearly two hours. It was abundant; sweating less prominent, showing itself especially between the shoulders. There was some nausea, but no vomiting. The sensibility and swelling of the salivary glands were tolerably well marked. Nothing peculiar as regards secondary actions. The same day the patient felt much better, the dullness diminished sensibly and the respiration was more free. Two days after the signs indicating renewed effusion, a second dose was given, and this time the effusion disappeared permanently.

The second case was a lady of 40 years. She had latent effusion on the right side of six weeks standing when she entered the hospital. Jaborandi was given in the same dose and manner. The effusion was greater than in the first case. He had resource to jaborandi four times during twelve days. Sweating and salivation were abundant. There was some nausea and vomiting, swelling of salivary glands, and hypersecretion from lachrymal glands, and nasal membrane. A third and similar case was also cured in the same manner by the author. The author remarks that the first dose was more energetic than following ones. Patients should avoid exposure to cold during the sweating. After the disappearance of the effusion the friction of the pleura was very distinct.

Prof. Combal thus formulates the action of jaborandi in pleuritic effusions:

1. Jaborandi is very useful in the treatment of pleuritic effusions of whatever age or however abundant the liquid.

2. Jaborandi causes most often the liquid to disappear very rapidly, and the friction sounds to be heard.

3. The effects of jaborandi are of short duration; the liquid often reforms with great rapidity. It is necessary then to insist upon the use of jaborandi, and usually the liquid may be made to disappear permanently.

4. But when once the liquid has disappeared and the friction sounds have appeared, jaborandi becomes absolutely useless. We must then, more generally, to accomplish a cure, resort to tonic treatment and sometimes to local applications of tincture of iodine for example.

M. Dujardin-Beaumetz has employed jaborandi with success in albuminaria, in which there was general œdema, continual vomiting, and scarcely 60 grams of urine passed a day. An infusion of 6 grams of the leaves in 90 grams of water was given by the rectum, and produced an abundant salivation and sweating. The patient was improved promptly.

Jaborandi also finds a use in asthma, especially in the humid variety.

OIL OF AMBER.—(*Phil. Med. Times*, Oct. 26, 1878.)

Dr. Finck relates several cases of angina and functional cardiac disturbances, especially those cases into which the neuralgic element enters, which yielded rapidly to the influence of the oil of amber. He gave it in doses of from four to ten drops, repeated every thirty or forty minutes during a paroxysm, or in much smaller doses for continued use along with other remedies. In hysterical angina, also, and in the breast, pang of deep agony or grief, eight or ten drops of the oil, with some ordinary antispasmodic remedy every twenty minutes, will often give the happiest relief.

